# Food Preservatives

**Published:** 1900-07-14

**Authors:** 


### FOOD PRESERVATIVES.

In discussing the question of the permissibility or otherwise of adding "preservatives" to substances intended for use as food, it is necessary to bear in mind that many such materials may act injuriously on digestion, even though they may not be of themselves directly poisonous. Much of the evidence given before the Food Preservatives Committee as to the non-poisonous character of some of the substances so used is then beside the mark, since it can be shown that they interfere with those preliminary digestive changes which food must undergo before it can be absorbed and made of use for the nourishment of the body. This was the drift of the evidence given by Professor Halliburton, F.R.S., of King's College, before this committee. The experiments' on which it was founded dealt more particularly with borax and formaldehyde. Their result was to show that one part of borax added to 1,000 parts of milk completely inhibits rennet activity, and that even smaller proportions delay its action. As to formaldehyde, the addition of the same proportion as is stated to be used in the milk trade, namely, two drops of formalin to the fluid ounce, greatly delays rennet action, and in some cases is sufficient to completely inhibit the operation of the ferment. Moreover, in no case is the curd which is produced of the same firm character as that which is formed in unaltered milk. The results in regard to the influence of weak solutions of formalin on the gastric and pancreatic digestion of proteids were equally instructive, such digestion being in some cases completely put a stop to. Of course, all these experiments were made *in vitro*, and require, perhaps, to be amplified *in vivo*. But artificial experiments on digestion have generally proved a pretty good guide to what occurs in natural digestive processes, and these results seem to prove sufficiently conclusively the injurious effects which are produced by even minute doses of certain preservatives on the activity of the enzymes concerned in ordinary digestion, and to furnish good grounds for prohibiting their addition to substances intended for the food of man.

[1] Brit. Med. Jour., July 7.

